# Review on the QM/MM Methodologies and Their Application to Metalloproteins

**DOI:** 10.3390/molecules27092660

**Published:** 2022-04-20

**Authors:** Christina Eleftheria Tzeliou, Markella Aliki Mermigki, Demeter Tzeli

**Affiliations:** 1 Laboratory of Physical Chemistry, Department of Chemistry, National and Kapodistrian University of Athens, Panepistimiopolis Zografou, 157 71 Athens, Greece; ctzeliou@chem.uoa.gr (C.E.T.); marmermigki@chem.uoa.gr (M.A.M.); 2 Theoretical and Physical Chemistry Institute, National Hellenic Research Foundation, 48 Vassileos Constantinou Ave., 116 35 Athens, Greece

**Keywords:** multiscale calculations, QM/MM, DFT, semi-empirical, molecular dynamics, molecular mechanics, metalloproteins, chemical reactions, nitrogenase, FeMoco

## Abstract

The multiscaling quantum mechanics/molecular mechanics (QM/MM) approach was introduced in 1976, while the extensive acceptance of this methodology started in the 1990s. The combination of QM/MM approach with molecular dynamics (MD) simulation, otherwise known as the QM/MM/MD approach, is a powerful and promising tool for the investigation of chemical reactions’ mechanism of complex molecular systems, drug delivery, properties of molecular devices, organic electronics, etc. In the present review, the main methodologies in the multiscaling approaches, i.e., density functional theory (DFT), semiempirical methodologies (SE), MD simulations, MM, and their new advances are discussed in short. Then, a review on calculations and reactions on metalloproteins is presented, where particular attention is given to nitrogenase that catalyzes the conversion of atmospheric nitrogen molecules N₂ into NH₃ through the process known as nitrogen fixation and the FeMo-cofactor.

## 1. Introduction

Warshel and Levitt introduced the multiscaling Quantum Mechanics/Molecular mechanics approach, i.e., QM/MM, for the investigation of complex molecular systems in 1976 [1]. This methodology was first applied to an enzymatic reaction. The extensive acceptance of this method started in the 1990s [2]. In this study, the conjunction of SE methods with molecular force field was completely illustrated, while the precision, and efficacy of the QM/MM treatment in opposition to ab initio and experimental data were estimated [2]. In the last few decades, a lot of simulations for biomolecular systems have been carried out using QM/MM approaches. Moreover, a lot of reviews evaluate these methods themselves and the updates that are established throughout the years. Additionally, this method is combined with others, such as methods that consider the quantum nature of atomic motion, including free-energy and reaction path methods for more accurate answers in studies of complex systems and especially in enzymatic reactions [3]. Generally, the QM/MM approach is established for modeling complex biomolecular systems, inorganic, organometallic, and solid-state systems, as well as for the study of processes that take place in explicit solvent [3].

In 2013, the Nobel Prize in Chemistry was awarded to Martin Karplus, Michael Levitt and Arieh Warshel equally, “for the development of multiscale models for complex chemical systems” as a reward for their significant contribution in computational chemistry. The theoretical calculations based on this theory can predict chemical processes, explain, and interpret experimental data [4]. Additionally, they are supplemental to the experimental information adding details. Karplus, Levitt and Warshel’s work is revolutionary because they combined the classical consideration of matter with quantum physics and chemistry. Until then, only one type of methodology had to be chosen, i.e., classical or quantum. Classical physics approached large molecules in a simpler way which was an advantage when calculating, counter to its weakness that is the incapacity of simulation of chemical reactions. On the contrary, the quantum consideration of systems can be applied only in small systems, since it demands enormous computing power. As a result, they could be applied for small molecules only. The QM/MM theory solves this impasse of choice, and it combines both theories for a more accurate simulation [4].

In the present review, new advances in the main methodologies, i.e., DFT, SE methods, MD simulations, MM, that are combined in QM/MM and QM/MM/MD approaches are discussed in short. Then, a review on reactions on metalloproteins emphasizing in nitrogenase is presented.

## 2. Methodologies

### 2.1. Density Functional Theory

DFT was introduced in 1964 by Hohenberg and Kohn [5]. It predicts the molecular properties based on the calculation of the electron density of molecules. The electron density of a molecule constitutes one of its physical properties. The DFT methodology contrary to the Hartree-Fock (HF) theory, where the full N-electron wavefunction is calculated, aims at calculating the total electronic energy by considering only the total electron density distribution. The inhomogeneous electron gas model suggested by Hohenberg and Kohn indicated that a system’s ground state energy, Ε, could be defined by its electron density, *ρ*(*r*). Specifically, the energy functional is written as follows:

(1) $$E\left[\rho \left(r\right)\right]=\int^{\;}V_{e/ext}\left(r\right)\rho \left(r\right)dr+F\left[\rho \left(r\right)\right]$$

where $\int^{\;}V_{e/ext}\left(r\right)\rho \left(r\right)dr$ corresponds to the interaction of the electrons with an external potential (e.g., the Coulomb interactions with the nuclei), while F[ρ(r)] corresponds to the kinetic energy and the contributions from interelectronic interactions. In 1965, Kohn and Sham [6] considered F[ρ(r)] to be a sum of three terms:

(2) $$F\left[\rho \left(r\right)\right]=E_{KE}\left[\rho \left(r\right)\right]+{Ε}_{H}\left[\rho \left(r\right)\right]+E_{XC}\left[\rho \left(r\right)\right]$$

where $E_{KE}\left[\rho \left(r\right)\right]$ is the kinetic energy of a system with non-interacting electrons with the same electron density as the real one, ${Ε}_{H}\left[\rho \left(r\right)\right]$ is the Coulomb energy of electrons, and $E_{XC}\left[\rho \left(r\right)\right]$ is a term that contains contributions from exchange and correlation energies while also corresponds to corrections in the kinetic energy that arise from the electron-electron interaction. In particular, the exchange energy is a stabilization energy that arises from the ability of same spin electrons to avoid each other. There is no classical analogue to this, and it comes from the Pauli principle. It is a stabilization energy since the real Coulomb repulsions are lower as the same spin electrons avoid each another. 

The major advantage of DFT is the inclusion of correlation energy. In a HF method, electron i is considered to move in an average potential that comes from the sum of electrons j, with i≠j. However, the motion of the electrons is instantaneously correlated, while they avoid each another in a more dynamic way than what described by an average potential, thus the real Coulomb repulsions between them are lower. Hence, correlation energy is a stabilization energy as well and it is included by the DFT method.

Thus, the full expression of the energy would be:

(3) $$E\left[\rho \left(r\right)\right]=\overset{N}{\underset{i=1}{\sum }}\psi_{i}\left(r\right)\left(\frac{-\nabla^{2}}{2}\right)\psi_{i}\left(r\right)dr+\frac{1}{2}\iint^{\;}\frac{\rho \left(r_{1}\right)\rho \left(r_{2}\right)}{\left[r_{1}-r_{2}\right|}dr_{1}dr_{2}+E_{XC}-\overset{M}{\underset{A=1}{\sum }}Z_{A}\int^{\;}\frac{\rho \left(r\right)}{r-R_{A}}dr\;$$

where the first term is the kinetic energy of non-interacting electrons system, the second one corresponds to the interelectronic repulsions, the third is the exchange-correlation energy and the last one is the Coulomb attractions between electrons and nuclei. Kohn and Sham considered the electron density to be the sum of the square modulus of *Ν* one-electron orbitals:

(4) $$\rho \left(r\right)=\overset{N}{\underset{i=1}{\sum }}|\psi_{i}\left(r\right){|}^{2}$$

The challenge of DFT is to find an appropriate functional to describe the exchange-correlation energy [7,8,9]. For this matter, several approximations have been proposed, leading to a plethora of functionals. Generally, there are four categories of approximations. 

The simplest approximation for the exchange-correlation functional is the Local Density Approximation (LDA). It is based upon uniform electron-gas and it presumes the uniformity of the molecule’s density all over the system. The local spin-density approximation (LSDA) is a generalization of LDA where the electron spin is included. Some of the most popular LDA functionals are the Vosko-Wilk-Nusair (VWN) [10] and the Perdew-Wang (PW92) [11] functionals. However, this approximation is not appropriate for molecules, wherein electron density is clearly nonuniform, while it works well for the calculation of the electronic band structures of solid-state. 

The second category contains functionals where a gradient correction factor is included; this category is a significant improvement to the LDA approach. The gradient corresponds to the non-uniformity that characterizes the electron density, and it is known as gradient-corrected (GC) or non-local functionals. Hence, *ρ*(*r*) is not considered to be constant. Typically, these gradient corrections are divided in separate exchange and correlation functionals, such as the Becke exchange functional B88 [12] or the Lee-Yang-Parr correlation function, LYP [13]. Their combination led to the widely used BLYP GGA functional. An improvement on the GGA is the meta-GGA approach, where the functionals depend on the density, on the gradient, on their second derivatives, i.e., M06-2L [14].

The third category includes the hybrid functionals, which seek to include some elements from ab initio methodologies along with improvements via DFT mathematical formulas. A percentage of precise HF exchange is included (i.e., ab initio exchange without any parametrization). This approach furnishes the hybrid GGA functionals, among which is B3LYP [15] (which contains a precise 20% HF exchange). Note that hybrid methods, for instance B3LYP, are preferred for computational chemistry calculations. Moreover, there are the hybrid meta-GGA functionals. One of them is the M06 functional which was proposed by Y. Zhao & D.G. Truhlar in 2006, and it contains a precise 27% of HF exchange [15]. At last, double hybrid functionals have been developed, such as B2BLYP, based on the meta-GGA approach for the inclusion of precise HF exchange, combined with a perturbative second-order correlation part acquired from the DFT orbitals and eigenvalues [16]. Finally, the recent range-separated functionals, i.e., HSE06, LC-wPBE, and RS-DDH belong in this category [17,18]

Finally, the last category includes combination of DFT with other ab initio methodologies, such as multiconfiguration pair DFT (MC-PDFT) [19], multireference DFT (MRDFT) method, [20] dynamical DFT (DDFT) which is an extended DFT approach to nonequilibrium systems [21] etc. However, all these DFT methods are more time-consuming than traditional DFT methods.

The generation of divergent types of functionals has been useful in describing a variety of systems and applications. It is important here to mention the functionals that include long-range corrections. Generally, the non-Coulomb part of the exchange-correlation functionals vanishes quickly and it is not accurate at large distances, making them unsuitable for the study of electron excitations to high orbitals, for non-covalent bonds as well as for van der Waals bonds which are typically found in biological systems. Various schemes have been constructed to handle such cases. Commonly used functionals for these cases are LC-wHPBE, [22], CAM-B3LYP, [23] wB97XD, [24], MN15, [25], etc.

Finally, the time-dependent DFT (TD-DFT) has been developed for the computing of the excited states of a molecular system. TD-DFT shares the same philosophy as DFT, but it considers a time-dependent problem [26]. According to the Runge-Gross theorem [27], the time-dependent electron density uniquely defines a time-dependent external potential at any time. The Hamiltonian of the system takes the following form:

(5) $$\hat{H}\left(t\right)=\hat{T}+{\hat{V}}_{e/ext}\left(t\right)+\hat{W}$$

Here $\hat{T}$ is the operator of the kinetic energy, ${\hat{V}}_{e/ext}\left(t\right)$ is the operator of a time-dependent external potential, $\hat{W}$ is the operator referring to electron-electron interactions. Within the TDDFT theory, initially a Kohn-Sham scheme is employed, in a similar fashion as the common DFT. So, a non-interacting system is considered, that yields the same time-dependent electron density with the real, interacting system. A Hamiltonian for the non-interacting system is constructed:

(6) $$\hat{H}\left(t\right)=\hat{T}+{\hat{V}}_{KS}\left(r,t\right)$$

${\hat{V}}_{KS}\left(t\right)$ stands for Kohn-Sham potential, which acts on the non-interacting wavefunction Φ(r,t).

In summary, DFT is a computational cheap methodology comparing to ab initio methods, such as multireference and coupled cluster approaches. It calculates a part of electron correlation energy which is determined as the energy of the exact solution obtained from Schrödinger equation minus the HF energy. However, contrary to other methods, decisions must be taken regarding to which functional will be used for a specific application. For instance, TPSSh functional [28] constitutes a very good choice for molecules including transition metals, but not for organic molecules. B3LYP is a good one, a “standard” one for projects involving relatively small closed-shell molecules, MPW1K [29], is an exceptional good one in studying modeling kinetics of reactions by determining the transition states, etc. Generally, although DFT is an accurate reformulation of quantum theory, approximations are needed regarding the Exchange-Correlation energy functional. Most of their deficiencies lies upon two main errors of standard density-functionals: the delocalization and static correlation error [30]. However, DFT approach is a computational cheap methodology comparing to ab initio methodologies, it can be used in systems up to a few hundred atoms, its accuracy can be compared to other ab initio methods, while efforts are being made to derive functionals suitable for many types of applications [25].

### 2.2. Semiempirical Methods

Semiempirical methods throw bridges across ab initio and empirical approaches when calculating large molecules of biological systems. These methods are built on the HF formalization with a lot of differences based on approximations and empirical data. They have been characterized as the new generation of SCF methods [31]. The main concept that SE methods follow is that some complicated integrals are not calculated, but instead they are parameterized and replaced by approximations. Thus, many terms which are not important can be neglected throughout this process. However, the errors resulting from the process, can be fixed by incorporating some empirical parameters into the very first formalism, while they are modified with respect accurate experimental or calculated reference values. The SE representation tries to maintain the crucial physics behind the studied system. The parameterization corresponds to all other effects in an average sense, ending with an evaluation based in numerical accuracy. Many times, these methods may not seem very accurate, but they are efficient at last [32].

The SE approaches are categorized in two major groups depending on their approach of the system. Firstly, we have the Hückel’s π-electron method, where MOs are generated basically from the molecule’s connectivity matrix. It is basically used for the calculation of the excited states of polyenes and other unsaturated molecules leading to some very important qualitative physical insights concerning the structure, stability, and spectroscopy [33]. An advance in this theory is the Hoffmann’s extended Hückel theory, where all valence electrons are included, which is robust for inorganic and organometallic compounds [29]. Below, various SE methods are reported, which have qualitatively improved the MO theory and they are used to understand some chemical phenomena in terms of orbital interactions [34].

In the first method group, the one-electron integrals are included and are therefore noniterative, while a two-electron integrals approach has been used, which appears to work in semiempirical SCF methods. Working on π-electrons, known as Pariser–Parr–Pople method, there is a successful approach for the electronic spectra of unsaturated molecules [35,36]. Moving to the second method group, Pople proposed a generalization to valence electrons and established approximations of integrals that content the rotational invariance and some other consistency criteria. In this group belongs the CNDO, INDO and NDDO methods. They stand for the complete neglect of differential overlap (CNDO), intermediate neglect of differential overlap (INDO), neglect of diatomic differential overlap methods (NDDO) [37]. Dewar recommended calibration against experimental reference data mostly for organic molecules at the ground state of their potential surfaces. Three new models were developed. The MINDO/3 method which is an INDO-based one, and the MNDO and AM1 methods which are NDDO-based ones. Next, with a new parameterization of the MNDO model, the PM3 was created. Officially, AM1 and PM3 are different from MNDO in the selection of the empirical core repulsion function only and are partly used to see the limits of parameterization of the MNDO electronic structure model [38,39,40,41,42,43]. There has been a generalization on the MNDO model from a sp basis to a spd basis. This can have legitimate results for heavier elements, particularly hypervalent main-group elements. Some extended parameterizations based on spd basis lead up to PM6 and PM7 methods, which are widely used, especially the PM6 method, for calculations of metalloproteins [44,45]. Lastly, a series of orthogonalization models, namely, OM1, OM2, and OM3, have been suggested where orthogonalization corrections are incorporated in the one-electron terms of NDDO [46].

Concerning each methodology development, it is based on different integral approximations and on the character of the interactions that are incorporated. For instance, MNDO and OMx handle the valence electrons via SCF-MO using a minimal basis set. The core electrons are calculated via reduced nuclear charge and electron correlation only if it is mandatory for zero-order description. Finally, dynamic correlation effects are subsumed through two-electron integrals and the general parameterization [32]. Integral approximations that are made based on the ignorance of all three-center together with four-center two-electron integrals, simplify the standard SCF-MO equations. These approximations are included in CNDO, INDO, and NDDO methods. Additionally, the MNDO-type methods that are used more in calculations of metalloproteins, use Slater-type atomic orbitals as the basis functions. After some adjustments, in the Fock matrix the one-center integrals have been exported from available atomic spectroscopic data. Some further parameterizations for one-center two-electron integrals and two-center two-electron integrals occur at large distances according to classical electrostatics. The original MNDO method parameterization emphasized on ground-state properties, mostly geometries and heats of formation, with the use of ionization potentials and dipole moments as supplementary reference information [32]. Ionization potentials together with dipole moments are also included as additional reference data. 

The AM1 and PM3 methods that are suitable for calculations of metalloproteins, are based upon the same model as the MNDO, but they vary from it in the effective atom-pair potential in the core–core repulsion function only. More adjustable parameters are included, making the basic function more flexible. The extra Gaussian terms are empirically used and not established in theory [41,42,43]. Lastly, the parameterization in AM1, PM3 and MNDO seem to have the same philosophy, but the optimization of parameters per element was increased to 18 in PM3, while was 5 to 7 in MNDO. The three methods use a sp basis without d orbitals and have no application to most transition metal compounds like a lot of metalloproteins. This issue is overcome when the two-center two-electron integrals are parameterized for a spd basis which is an extension of the original point-charge model for a sp basis. Note that this is used in MNDO/d extension which can be applied in conjunction with any MNDO-type approach like the PM6 and PM7 methods. These methods are widely applicable since they have been parametrized for many elements, i.e., the MNDO/d parameters are applicable to second-row elements, halogens, and zinc group elements. Specifically, the PM6 parameters have been established for most Periodic Table elements, specifically for about 70 elements so far [32].

To conclude, SE methods are valuable tools for studying electronic effects in large molecules and they can be applied successfully in complex systems. More details for the above formalisms can be found in in the original publications and several comprehensive review articles [47,48,49,50,51,52,53,54].

### 2.3. Molecular Mechanics (MM)

The investigation of processes of biological importance often requires modelling of large systems, consisting of hundreds or thousands of atoms. The number of electrons present in such systems is too demanding for rigorous quantum chemical calculations, even for the current computational capacity. Thus, the empirical MM methodology is employed, under which the energy of the system is considered with classical mechanics. Note that the Born-Oppenheimer approximation is also considered. The potential energy of the system is determined as a function of the nuclear coordinates with the use of molecular force fields (FF), while the motions of electrons are not considered. The energy of the system is described by its Hamiltonian, but within a classical (Newtonian) framework. It includes a kinetic energy term as well as a potential energy term: ℋ=K+V. However, for the definition of the potential energy V, special attention is needed. As reported above, the potential energy of the system can be defined with the use of the force field method, where electronic motions are not considered, and the energy of the system is determined as a function of nuclear positions only. The molecular force fields can be regarded as a rather simple, four-component picture of the intra- and inter-molecular forces inside a system. Specifically, the system’s potential energy [55,56,57] can be analyzed as

(7) $$V\left(r^{N}\right)=\underset{bonds}{\sum }\frac{k_{i}}{2}{\left(b_{i}-b_{o}\right)}^{2}+\underset{angles}{\sum }\frac{k_{i}}{2}{\left(\theta_{i}-\theta_{o}\right)}^{2}+\underset{torsions}{\sum }\frac{V_{n}}{2}\left(1+\cos\left(n\omega -\gamma \right)\right)+\overset{N}{\underset{i=1}{\sum }}\overset{N}{\underset{j=1+1}{\sum }}\left(4\varepsilon_{ij}\left[{\left(\frac{\sigma_{ij}}{r_{ij}}\right)}^{12}-{\left(\frac{\sigma_{ij}}{r_{ij}}\right)}^{6}\right]+\frac{q_{i}q_{j}}{4\pi \varepsilon_{o}r_{ij}}\right)$$

where the first term is a summation over all the bonds, the second a summation over all the angles, the third a summation over all the torsions, and the fourth includes all non-bonded interactions, i.e., van der Waals (vdW) and Coulomb contributions. 

*Bond terms*: Molecules undergo vibrational motion which is modelled as a harmonic potential, according to Hooke’s law [57]: $v\left(b\right)=\int^{\;}F\left(b\right)db=\int^{\;}-kb=\frac{k}{2}{\left(b-b_{o}\right)}^{2}$, where k represents the force constant, $k=\omega^{2}\mu$, ω is the vibrational frequency of the bond, μ the reduced mass and $b_{o}$ the equilibrium value around which a bond oscillates. Both k and $b_{o}$ are parametrized for the type of atom that participates in the studied bond. In most force fields, an atom type contains additional data concerning its hybridization state and even the local environment [53]. Although modelling a bond using Hooke’s law allows for some vibrational deviation from the equilibrium bond length, the true bond-stretching is not harmonic. Due to this non-harmonic nature, its average value will deviate from the equilibrium bond value and in high energies it would even be dissociative. Nonetheless, Hooke’s law functional form is a logical approach at the equilibrium bond distances of the ground-state molecules, a more accurate approach is the use of the Morse potential: $v\left(b\right)=D_{e}{\left(1-e^{\left[-\alpha \left(b-b_{o}\right)\right]}\right)}^{2}$, $D_{e}$ is the depth of the potential energy minimum, $\alpha =\omega \sqrt{\frac{\mu }{2D_{e}}}$, *μ* is the reduced mass and *ω* is the vibrational frequency of the bond. Although the bond is described more accurately, the Morse potential is not usually used in MM force fields, since it requires three parameters to be specified for each bond. The inability of modelling a bond break (and a bond formation, respectively) is amongst the most important restrictions of the MM methodology. Thus, the QM methodology must be employed to consider, examine, and interpret these phenomena. 

*Angle terms*: The deviation of angles from their reference values is described using a harmonic potential. The second term of potential energy V, $\underset{\mathrm{angles}}{\sum }\frac{k_{i}}{2}{\left(\theta_{i}-\theta_{o}\right)}^{2}$ describes a bond bend, which is also characterized by a force constant $k_{i}$ and an equilibrium angle $\theta_{o}$. Both are distinct for each type of atoms and for their characteristics such as their hybridization.

*Torsion terms*: They are included to describe the steric barrier between atoms separated by three covalent bonds. The motion associated with this steric effect is the bond rotation described by a dihedral angle around the bond connecting the two middle atoms. Unlike bond stretches and bends, which require quite substantial energies to cause significant deformations from their reference values, dihedral bends are less energetically expensive and correspond to most of the variations in structure and relative energies of a molecule. The third term of potential is regarded as a periodic one, $\underset{\mathrm{torsions}}{\sum }\frac{V_{n}}{2}\left(1+\cos\left(n\omega -\gamma \right)\right)$, and it represents the rotational degrees of freedom of the molecule, where *V_n_* is a constant corresponding to the barrier height of rotation, n is the multiplicity, specifically, the number of minimum points of the function as the bond rotates over 360° corresponding to the periodicity of the function, *ω* is the dihedral angle, while *γ* is the phase factor, which determines the point where the torsion angle passes through its minimum.

*Non-Bonded Terms*: They consist of the vdW and Coulomb contributions. In most force fields, vdW contributions to the potential energy are described by the Lennard-Jones potential [58]: $V_{vdW}=\overset{N}{\underset{i=1}{\sum }}\overset{N}{\underset{j=1+1}{\sum }}\left(4\varepsilon_{ij}\left[{\left(\frac{\sigma_{ij}}{r_{ij}}\right)}^{12}-{\left(\frac{\sigma_{ij}}{r_{ij}}\right)}^{6}\right]+\frac{q_{i}q_{j}}{4\pi \varepsilon_{o}r_{ij}}\right)$, where *r* refers to the distance connecting two particles, *ε* is the depth of the energy well and *σ* is the interatomic distance, for which the energy becomes zero. Distance *σ* represents the minimum distance at which two particles can approach each other because for r<σ the potential energy tends to infinity, while in long distances, the potential energy tends to zero, i.e., the particles do not interact. Finally, Coulomb interactions are described by the Coulomb’s law.

To determine the functions as well as the parameters which include the FF, atom types are used by MM. As mentioned above, an element may be determined by various MM atom types, depending on several characteristics and conditions, for instance hybridization and chemical environment. Some examples of MM force fields are UFF, Dreiding, MM2, MM3, MM4, MMFF, AMBER, CHARM, OPL, and ECEPP. UFF considers the type of element, its hybridization, as well as its connectivity. Additionally, UFF can be employed in MD simulations. Dreiding employs general force constants along with geometry parameters, while hybridization is considered. MM2 is used mainly for simple organic molecules, i.e., ethers, ketones, aromatic compounds, etc. In MM2, the anharmonic breakage of bonds is included via additional terms. MM3 is an improved form of MM2 including potential functions, where corrections and/or modifications, i.e., correction of high rotational barriers in congested hydrocarbons, alternations in vdW parameters etc, are considered. MM4 incorporates some interactions, such as torsion–bend along with bend–torsion–bend interactions, resulting in a better calculation of vibrational frequencies. MMFF (Merck Molecular FF) comprises of a broad range of excellent data used for MM and MD simulation. AMBER stands for Assisted Model Building with Energy Refinement. It is appropriate for the modeling of both small molecules and polymers. CHARMM stands for Chemistry at HARvard Macromolecular Mechanics, and it is appropriate for application such as conformational analysis, molecular minimization, free energies. It is applied in the study of biomolecules, i.e., peptides, nucleic acids, proteins, lipids, and carbohydrates. OPLS stands for optimized potentials for liquid simulations; it employs additional functions that denote the H-bonding. Finally, the ECEPP, which is an empirical conformational energy program for peptides, uses experimental data that are continuously updated, and it employs a series of parameters for the definition of the geometry of amino acid residues and the interatomic interactions. To sum up, there is a series of molecular force fields suitable for various applications, so as the investigation of chemical processes involving large systems to be feasible in a good level of accuracy.

### 2.4. Molecular Dynamics Simulations 

MD simulations are very useful for the understanding of the physical basis of the structure and the functions of many biological macromolecules. They were first developed in the late 70s and they have evolved throughout the decades from a simulating system of hundreds of atoms to macromolecules of biological interest such as nucleosomes, ribosomes, and the macromolecules of our interest, metalloproteins. The range of the population of atoms of the calculated systems varies from 50,000 up to 500,000. The most populous systems need appropriate computer facilities, and it can be succeeded using high-performance computing (HPC). A dynamic model is built for metalloproteins where the internal motions as well as the resulting conformational changes are both important in their functions. This is contrary to the old considerations that the proteins have a rather rigid geometrical structure [59].

MD simulations depict and predict the trajectories of the particles of a studied system. To accomplish this calculation correctly, a simple algorithm is developed, which calculates the trajectories through force field approach. It begins with the potential energy calculation *E_pot_* {*x_i_*} of every particle, it continues with the calculations of the acting forces *F_i_* = −*E_pot_*/*x_i_* and the acceleration *a_i_* = *F_i_*/*m_i_* of it. It ends with calculating the velocity *v_i_* (*t* + d*t*) = *v*(*t*)*_i_* + *a_i_* dt and the particle coordinate *x_i_* (*t* + d*t*) = *x*(*t*)*_i_* + *v_i_* d*t*. In this way, we result in a complete trajectory of a particle. The algorithm works for 3N particles in total [59].

Moving on, the representations are based to different levels of details. The metalloprotein can be initially modelized using structures found experimentally or from other modeling data. The atomistic representation is not commonly used for large systems such as metalloproteins, even though it leads to the best reproduction of a system. The most suitable model is the coarse-grained (CG) method, where molecules are represented by “pseudo-atoms” approximating specific groups of atoms, for example they represent the whole amino acid residue and individual atoms are not considered [60]. At first, CG models were developed based on classical statistical mechanics, but with the passage of time, CG methods considering quantum Boltzmann statistics were established [61]. Specifically, scientists try to reproduce information from a fine-grained atomistic level to CG approaches for a better description of the studied system based on natural laws. A bottom-up CG theory in quantum Boltzmann statistics based on the Multiscale CG methodology has already been developed, which describes more accurately biomolecular systems [61]. Additionally, other bottom-up evolved methodologies are developed that are based on inversion of Monte Carlo simulation, Boltzmann Inversion and iterative variation and multiscale CG methodology generally [62,63,64,65]. Regarding the solvent, it can be calculated with metalloproteins. The solvent representation constitutes a crucial matter for the system under investigation. Of course, the addition of solvent molecules explicitly is the most effective approach, and the success of this representation is influenced by the increase in the size of the system. The explicit solvent addition can retrieve most of the solvation effects together with those that result from entropy such as the hydrophobic effect. While all their ingredients and the approaches of them have been discussed, the interactions of them are studied through force-fields. All above lead to the calculation of the potential energy of the system under study [59].

The force-field representation includes solutions of complex equations which occurs easily with the assistance of computer systems. The bond length and the angles are represented by springs. Periodic functions are used for bond rotations and Lennard–Jones potentials, along with Coulomb’s law which is used for vdW and electrostatic interactions, respectively. In this way, energy and force calculations, even for large systems, are exceptionally rapid. FF that are used in atomistic molecular simulations are parameterized in a different way. This is a consequence of different types of FFs, namely general FFs that are used widely for various chemical compounds and dedicated ones for specific types of systems [66]. For a suited selection of FF for a studied system, the desired results need to be taken into consideration, i.e., FFs based on spectroscopic, structural, and thermodynamic data are chosen in calculations of analogous properties. Combining an accurate reproduction of the desired structure with the right FF, there will be a correct approach of the studied system [66]. As the parameter fitting is concerned, cautious selections of potential energy functions set, reference data set and a methodology for quantitatively correlating experimental and calculated structural parameters, are essential for a successful computational representation. Most parameter fitting is succeeded manually, but there is a development of automatically optimizations. Lastly, the parameters that may be chosen, may originate from both experimental and theoretical data. The most common and more suitable parameters are got from structural data from X-ray experiments, but in occasions where there are no experimental data, structural parameters are extracted from DFT calculations [66]. Simulations, where modern FFs are used, are commonly equal, but parameters used in classical FFs representations are not definitely exchangeable, while not all FFs permit representation of all molecule types [67,68]. A representative case is the different conclusions for helicity of proteins structures that are obtained from a classic set Amber ff14SB in conjunction with TIP3P three-point water model and standard ions compared to the modern set Amber ff19SB with OPC four-point water model and 12-6-4 ion parameters. The classic set approach results in an inherent underestimation of helicity in a protein structure counter to the modern set, which appears to have better predictive power not only in the basic protein structure, but also for protein mutations, sequence-specific behavior, and rational protein design [69]. Despite all these, when studying a reacting system such as reactions of metalloproteins, a Reactive FF needs to be used for the best description of the system. Therefore, a lot of reactive FFs have been developed. Specifically, the ReaxFF is the most common one, where Coulomb and Morse (van der Waals) potentials participate in calculations of nonbond interactions between all atoms of the reactants [70]. Parameters need to be derived from already verified calculating methods such as calculations on bond dissociation and reactions of small molecules combined with formation heat and structural data [70]. Adding to that, another widespread reactive FF is the Empirical Valence Bond (EVB) model, wherewith chemical reactions that are carried out by enzymes or in condensed phases are truthfully studied in different environments [71]. Moving on, the law of motion from classical physics is employed for the calculation of accelerations and velocities and for the update of the atom positions in all FFs types. The use of a time step shorter than the fastest movement in the molecule is essential to avoid instability when the integration of movement is completed numerically. One of the most significant obstacles in this simulation procedure is the fact that this integration ranges usually between 1 and 2 fs when referring to atomistic simulations. The microsecond-long simulations for biological processes, demand 109 times repetition over this calculation cycle. This constitutes one of the strengths of the coarse-grained approaches. The time length of the simulations is expanded when the system is represented in a simpler manner, and thus more time steps occur. For the increase in the performance of MD simulations, algorithmic advances are used, i.e., parallel running, graphical processing units, namely, GPUs, and fine-tuning of energy calculations [59].

Nowadays, the new generation computers are equipped and supported with accelerator and the parallelism process which are suitable to fasten the simulation. Specifically, the simulation codes AMBER [72], CHARMM [73], GROMACS [74], or NAMD [75], that are the most commonly used, are running in parallel via messaging passing interface (MPI). MPI is very suitable for reducing the computation time in the case where many computer cores are used at the same time. With the aim of exploiting the locality of interactions, the system is distributed to processors. The term for this scheme is spatial decomposition and each processor is used to accommodate the simulation of a small part of the system solely. Each processor is responsible for simulating a space region independently of the total number of particles, leading to the most profitable partition when simulating is based on position in space of the particles that are included in the studied system. Additionally, the processors are not sharing information between each other, except when they are simulating neighboring regions of the simulated system [76]. A breakthrough in simulation codes is the use of accelerators like GPU. They represent a great technological advance in performing atomistic MD calculations. So far, most important MD codes have been developed for GPUs, while MD codes have been constructed especially to be used on GPUs (ACEMD [77]). In order to achieve a high performance of MD simulations, they run on GPUs, and sometimes they are adjoined with MPI. Closing, the HPC use in natural and life sciences is developing more and more now. The improvement of their performance leads to more accurate simulations with the help of increasing power and sophistication of GPUs.

### 2.5. QM/MM and QM/MM/MD Approaches 

During a chemical process, the electronic structures of the involved species can alter. For instance, bonds are broken or formed. As a result, the inclusion of the electronic motion is required, i.e., a quantum mechanical description is required. For instance, for the study of a chemical reaction in a solvent, a QM methodology has been proposed for the molecular species, while the solvent is included via a dielectric constant, i.e., it is modeled by presuming a homogeneous polarizable medium [78,79,80,81]. However, as the magnitude of the employed QM system is increased, the selection of a dielectric constant is less significant, while the choice of the correct dielectric constant is not trivial [80,81]. Note that all-important energetic components, i.e., solvation and dispersion and all structural components, involved in a direct or indirect way need to be included [82]. Finally, it is known that some of the solvent molecules that interact with the studied molecular system must be treated explicitly, i.e., they should be included in the QM calculation [83]. 

Nonetheless, this approach is attainable for systems having not more than about several hundred atoms. For larger systems, the only solution is a multiscaling approach, i.e., a combined QM/MM approach, where QM is used to treat the main part of the system. At the same time, classical force field methods are usually employed for the rest [84,85,86,87,88]. Here, the most crucial task is to have an efficient interface between QM and MM, where four important features must be taken into consideration: (i) the partitioning of the system into QM and MM parts (ii) how the interaction between MM and QM is dealt with, (iii) how the covalent bonds between atoms at the QM/MM boundary will be calculated, (iv) how the total energy will be computed [82,89]. Finally, the dynamics of the system is important to be incorporated. For instance, it could be included via an MD approach, which calculates time averages of equilibrium properties. Note that, simulations are usually at the minimum 10 times longer than the slowest studied natural process [89,90,91,92,93]. Additionally, there are two other crucial aspects which are raised, specifically, the simulation protocol as well as the splitting of the system into MM and QM regions which is kept fixed during the simulation. Below, the four essential aspects, that must be considered for an efficient boundary between the two types of regions, will be analyzed. 

*Partitioning*: Regarding the study of a chemical reaction in solution, the partitioning of the system into QM and MM parts and its drawbacks will be explained. For the QM system, there are two alternatives: (i) only the solute molecules (this approach has been discussed above), (ii) the solutes and the nearest solvent molecules will be included. It has been mentioned that the latter choice is better, but there are some issues: the neighboring solvent QM molecules at the start of the simulation are replaced by MM solvent molecules during the MD simulations [94], and the solute−solvent interactions are not included accurately. Thus, the QM solvent molecules need to be kept near to the solute. A simple treatment of this solvent exchange issue is the update of the solvent molecules which are treated as QM or MM according to their relative position with respect to solute molecules. However, this treatment results in spatial and time-related discontinuities. The first ones result from the artificial boundary between the two regions, QM and MM. Note that there are differences concerning solvent properties at QM and MM levels and this results in an instability to the MD simulation. Thus, two different approaches are employed to solve these issues, namely, the constrained and the adaptive QM/MM [82,94]. The constrained QM/MM approaches derive from a single QM/MM partitioning scheme, i.e., BCC, BEST, FIRES [95,96,97]. The boundary between the two regions is closed and the QM solvent molecules stay fixed during the simulations, but now the deriving dynamic is not realistic. Thus, the constrained approaches are used only to reproduce equilibrium properties and are incapable of being employed for the study of reactions or diffusion dynamics. On the contrary, adaptive QM/MM approaches are open-boundary, i.e., they permit the smooth exchange of solvent molecules between the two regions depending on their distance from the solutes. Thus, they can study both equilibrium properties and dynamics [98,99,100,101]. Finally, it should be noted that the adaptive QM/MM methodologies usually are formulated on multi-partitioning schemes, i.e., different partitioning schemes are regarded [100,101].

*Interaction between QM and MM regions*: There are three ways to approach the electrostatic interactions between these two regions: (i) electrostatic embedding, which activate the polarization of QM region; (ii) mechanical embedding, which is less accurate than the first one, and it considers the atoms in QM region as point charges, bond dipoles, or higher multipoles; and (iii) polarizable embedding, which regards the polarization of the MM part as a reaction to the charge distribution [102].

*Crossing of the covalent bonds connecting atoms at QM/MM boundaries*: There are various opinions and options. For the crossed covalent bond in the boundary of the two regions, link atoms, pseudoatoms, or localized orbitals are introduced [1,102,103,104].

*Total energy*: It can be calculated using an additive or a subtractive QM/MM coupling [105,106]. (i) Additive QM/MM approach: The QM system is embedded within the MM one. The total E energy is, E = E_QM_ + E_MM_ + E_QM/MM_. Here, E_QM_ refers to the energy related to the QM region, E_MM_ to the energy of the MM region, while E_QM/MM_ corresponds to the interaction between QM and MM subsystems and contains the bonded interactions (QM/MM coupling terms). (ii) Subtractive QM/MM approach: Here, an extrapolation from a QM part to the whole system is conducted out. The total energy E is, E = E_QM_^(QM)^ + E_QM/MM_^(MM)^ − E_QM(MM)_. E_QM(QM)_ is the energy of the QM region computed at QM level, E_QM(MM)_ is the energy of the QM region computed at MM level, E_QM/MM_^(MM)^ is the MM energy of the whole system.

Synoptically, the combination of the QM/MM methodology with direct MD simulation, is a robust tool for studying drug delivery, chemical reactions mechanism in a complex environment, properties of molecular devices, organic electronics, etc., [59,94,95,96,97,98,99,100,101,102,103,104,105,106,107,108,109,110,111,112,113,114]. An analytic computational protocol for a multiscaling modeling of enzymes is given in [82]. Finally, it should be mentioned that the growing development of computing power and capacity allows us to study very large and complicated systems, to study their properties and to explain many complicated processes.

### 2.6. Computational Times of Methodologies 

Generally, the QM methodologies can be very accurate for small systems. However, they are computationally expensive, and as the size of the system is increased, their computational time is increased sharply. On the contrary, the MM methods are much faster, but they suffer from several limits, such as their requirement for extensive parameterization and the fact that the calculated energies are not very accurate. QM/MM approach constitutes a new class of efficient methodologies that combines the good points of both methodologies, i.e., the accuracy of QM and the speed of MM calculations. The most important advantage of hybrid QM/MM method is the speed. The cost of doing classical MM in the most straightforward case scales is O(N^2^), where *n* is the number of atoms in the system, meaning that a system having twice many atoms it would take four times as much computing power. This is mainly due to the electrostatic interactions term. Moreover, via the use of cutoff radius, periodic pair-list updates, and variations of the Particle Mesh Ewald (PME) method, computational time ranges from O(N) to O(N^2^). On the other hand, the simplest ab initio calculations typically scales is O(N^3^), while the accurate coupled cluster singles + doubles + perturbative triples RCCSD(T) methodology scales up to O(N^7^) [115,116]. To overcome this limit, a small part of the system is treated quantum-mechanically using a cheap QM methodology such as DFT, and the remaining system is treated classically. In more sophisticated implementations, QM/MM methods treat both light nuclei susceptible to quantum effects (such as hydrogens) and electronic states. This allows generating hydrogen wave-functions. This methodology has been useful in investigating phenomena such as hydrogen tunneling [116]. 

## 3. Metalloproteins

Μetalloproteins are proteins having a metal ion cofactor [117]. Metalloproteins can be found in many living species. It is regarded that half of all recorded proteins consist of a metal compound, while the metal compounds play a determinative role in their function in some of these cases [118,119]. Metalloproteins have a variety of functions, i.e., they storage and transport elements that are significant in a cell’s living or they transport even larger molecules. One of the most important functions is the catalysis of various chemical reactions that occur in a cell’s environment [117,120]. The most common metal elements found in the metalloproteins of a human body are Fe, Mn, Zn, Co, Ni and Cu, and they are considered to be of vital importance. However, the metals are not always a part of the active center of the protein or assist the protein’s involved processes. Thus, they can just be carried and transported by the protein [121].

There are two major groups of reactions related to metalloproteins. First, there are reactions which lead to the formation of metalloproteins. This group seems almost too complicated to be studied by a MM/MD simulation and there is limited literature on this topic. The second group of reactions, which is more often studied via MM/MD simulations, includes reactions that occur when the metalloprotein acts as a reactant or a catalyst. The increase in computational capacity and the theoretically development of simulation approaches in conjunction with the experimental data (crystallography) have resulted in further clarification of the way that metal clusters are assembled or inserted into target proteins. Additionally, the catalytic pathways of such a range of complex chemical reactions by metalloproteins is clarified and explained [122].

The computational characterization of metalloproteins can be an exceptionally difficult task. The presence of a metal cations is responsible for strong Coulomb forces that act on charged amino acids and the rest of the molecule. Proteins respond dramatically to the insertion or extraction of metal cations. Significant conformational modifications are observed and even aggregations occur. Metals having partially occupied d atomic orbitals favor specific coordination geometries. Regarding the metal, the geometry of the whole molecule and the dynamics of the surroundings and of the environment may or may not favor these coordination modes. Variations from the desired geometry decrease the protein-metal binding affinity. Note that, the electronic structure of a metal is directly affected from its surroundings. The electronic configurations of the metal depend on its ligands. Thus, the metal’s electronic structure and geometry of the molecule are strongly related with each other. Any modification of each one causes changes to the other one [123,124,125,126,127,128,129,130,131,132].

In this review, we are going to focus on important computational studies using different approaches which have been conducted for some vital metalloproteins, while attention is given to nitrogenase and its FeMo cofactor. 

### 3.1. Reactions of Metalloproteins

DFT approach usually is employed for a quantitative estimation of the complexation energies of several transition metal cations. The selectivity of metal-binding sites is investigated calculating the interaction energies between cations and its environment. Simple molecules with a general formula [MX_n_]^a+^ (where Xi’s are simplified ligands representing the protein environment) are studied and the energies of the transition metal ion complexation are evaluated. When small and large representations of metal-binding sites, i.e., small and large L ligands, are compatible with each other, useful information for reaction in even bigger systems are provided, see for instance [124,125]. 

The effect of specific groups or bonds on the properties and functions of proteins are studied also by using simplified ligands. For example, in the case of oxymyoglobin, which is a single chain globular protein, the hydrogen-bonding effect on Mössbauer spectroscopic properties is studied, for various active site models [125]. A porphyrin is used for representation of the heme group, and it is found that the H-bond between an His residue and the diatomic O_2_ enhances the binding of oxygen in the active center of protein [125].

It should be noted that metalloproteins’ metal centers present versatile chemical reactivity. The use of single-molecule atomic force microscopy (AFM) induces partial unfolding and exposes the metal centers. The rubredoxin is the first metalloprotein that has been studied via single molecule AFM in detail. QM/MD calculations on rubredoxin descripted in detail its unfolding and the breaking mechanism of ferric–thiolate bonds in different solvent conditions [132]. 

QM/DMD (discrete MD) approach works through a repetitious approach between QM and DMD [126,127]. DMD is a simplified MD, where discrete step function potentials are employed in the place of the continuous potential which are employed in common MD. Thus, the ballistic equations of motion are solved only for the species participating in a collision. In all, the QM/DMD predicts the structures of the metalloproteins, in agreement with X-ray experiment, as well as specific structural details, such as bond lengths of weak hydrogen bonds and their variations upon mutations in the protein. The method also can reintroduce the protein’ structure to equilibrium after a mild distortion due to the property of the combined potential energy function reaching its minimum at the intrinsic structure [123]. Up to now, it has been successfully used for the study of the function of ARD (acireductone dioxygenase) enzyme, which catalyzes two different oxidation reactions, depending only on which ion is bound to the protein, Fe^2+^ or Ni^2+^. The interconversion between the Fe^2+^-ARD and Ni^2+^-ARD is simple. Both forms of ARD were found that have different functions and the QM/DMD approach was an ideal methodology for the study of this interconversion [127]. Additionally, it has also successfully used in the modeling of the ion exchange, Ca^2+^ versus Mg^2+^, in the catechol-O-methyl transferase (COMT) enzyme, in the Fe-S electron-transporting protein rubredoxin and in several of its mutants [123].

Furthermore, in some proteins, the metal replacement can result in large-scale changes in geometry, protein motions and repacking, as is the case of COMT enzyme. COMT is enzyme involved in the physiology of pain. COMT has a Mg^2+^ cation, which can be interchanged with a variety of cations. This replacement results to significant alters in the structure and the activity of the enzyme. It influences the catalytic function, suppress it or it turns the enzyme to be an inhibitor. The inhibition is found that it is a simple geometric result. Multi-scaling calculations explains all mechanistic paths [127].

The metal-MFCC approach, namely metal molecular fractionation with conjugate caps, has been developed for efficient linear-scaling QM calculation of the potential energy and for atomic forces of metalloproteins. The protein’s potential energy is computed as a linear combination of (i) the potential energies of the neighboring residues, (ii) the 2-body interaction energy between non-adjacent residues, which are closely located, and (iii) the potential energy of the metal binding group. Each individual fragments in metal-MFCC can be calculated independently, so as the approach to be suitable for massively parallel computations. Thus, as the size of the studied system is increased, the computational cost of the QM calculation for the whole system increases rapidly. On the contrary, the computational cost of the metal-MFCC method increases almost linearly. It has been found that the metal-MFCC is in good agreement with full QM approach [128].

Recently in 2021, multiscale quantum refinement methods, combining several multiscale computational schemes with experimental data obtained from X-ray diffraction, were developed for metalloproteins. Different ONIOM combinations of QM, SE, and MM methodologies were used to check the performance and reliability on the refined local structure in two specific metalloproteins. It was found that ONIOM (QM/SE/MM) approach presented good results with low computational costs compared to the more expensive QM/SE approach [129]. This approach takes advantage of different flexible ONIOM schemes and experimental (XRD) information, in which the demanding transition-metal binding site is described with an efficient and accurate QM method, while the remaining system and its interactions are approximated by much faster computational low-level methods. Thus, this QM/SE/MM approach was proposed as a very good choice for computation of metal binding site(s) in metalloproteins with high efficiency.

Gallium cation, Ga^3+^, can mimic the ferric ion, Fe^3+^, and as a result it intervenes to some processes in which ferric cofactors are required. Thus, Ga^3+^ as a salt is used to fight various types of cancer and infectious and inflammatory diseases. However, they present some differences, for instance, Ga^3+^ ion cannot participate in redox reactions, or it has a different ability regarding the deprotonation of the bound water in aqua complexes. In summary Ga^3+^ and Fe^3+^ are distinguishable for some biological processes. The interactions of cations with protein ligands play a key role in their competition. These systems have been calculated via DFT, while the surroundings were represented by an effective dielectric constant. The DFT results explain and confirm the experimental findings, while they result in significant conclusions regarding the binding affinity of cations with respect to the change of the pH and of the environment [130].

The electron-transfer rates and the electronic-coupling interactions in proteins have been calculated and compared with available experimental data for a series of ruthenated azurins [131]. The DFT data are in good agreement with the experimental ones. The conformers with the strongest electron-coupling dominate on the electron-transfer rate, while the averaging, over all thermally accessible conformers of the protein and of the redox cofactors, is crucial. It is concluded that electronic coupling values based on calculations reproduce the coupling-limited experimental rates when the rates are averaged over ligand-field states and thermally accessible geometries [131]. 

Many studies regarding the use of MD and QM/MM in metalloproteins have been conducted. For instance, a combination of docking, QM/MM methods, and MD simulation has been used for binding affinity estimation of metalloprotein ligands [133]. Additionally, heme-containing proteins, due to their physiological importance, have been extensively characterized by computational methods and were the first protein class to be studied by MD simulations with Karplus’s work on myoglobin [134]. QM/MM calculations with DFT have been carried out for considering protein effects on the EPR and optical spectra of metalloproteins. Here, plastocyanin was used as a case study [135]. The QM/MM method has also been used to assess metalloproteins, human deacetylases, which are targets for a variety of medical conditions including neurodegenerative diseases and HIV infection. The method has also been proved to be capable of describing the kinetic differences associated with replacing Zn^2+^ with other metal co-factors [136]. In another case, the key step in the reaction mechanism of multicopper oxidases—the cleavage of the O–O bond in O_2_—has been investigated using QM/MM methods [137]. In general, enzymatic reactions have been the primary target of QM/MM studies. The examples of chorismate mutase and cytochrome P450 have been highlighted. Chorismate mutase catalyses the Claisen rearrangement of chorismate to prephenate, a key step of the shikimate pathway for the synthesis of aromatic amino acids in plants, fungi, and bacteria. On the other hand, cytochrome P450 enzymes are monooxygenases that perform a variety of essential functions, such as detoxification and biosynthesis, in nearly all living species. They also catalyze many types of reactions [138]. QM/MM reaction pathway analysis has provided detailed insight into the chemistry of glutathione S-transferase and can be used to obtain mechanistic insight into the effects of specific mutations on this catalytic process [139]. A developed QM/MM modification of the Linear Response method was used to distinguish ligand affinities for closely related metalloproteins. The precision level acquired makes the approach a useful tool for design of selective ligands to similar targets, as results can be extrapolated to maximize selectivity [140]. A QM/MM study of the formation of the elusive active species Compound I of nitric oxide synthase from the oxyferrous intermediate showed that two protons should be provided to produce a reaction that is reasonably exothermic and that leads to the appearance of a radical on the tetrahydrobiopterin cofactor [141]. QM/MM calculations have been employed to investigate the role of hydrogen bonding and π-stacking in single- and double-stranded DNA oligonucleotides [142]. MD simulations of metalloproteins were also carried out in a folding study of rubredoxin from Pyrococcus furiosus [143].

### 3.2. Nitrogenase and FeMo Cofactor

#### 3.2.1. General about Nitrogenase—Structure

Nitrogenase is one of the most fascinating natural metalloenzymes. It is produced by certain prokaryotes, such as cyanobacteria and it is essential for all living beings. Nitrogenase catalyzes an essential step of procedures in nitrogen fixation, where the reduction in the N_2_ to NH_3_ occurs through a complex and multistage reactions [144,145,146,147,148,149,150,151,152,153,154,155,156,157,158,159,160,161,162,163,164,165,166,167,168,169,170,171,172,173,174,175,176,177,178]. Ammonia is vital for all species, because of its essential role in synthesis of biomolecules such as nucleotides and amino acids. Despite the fact that N_2_ is abundant in the earth’s atmosphere, it is essentially inert at room temperature without a suitable catalyst. That leads to the vital role of nitrogenase. As a result, the scientific community is highly interested to study properly this reaction both through experiments and simulations. It is known that Nif genes or homologs have the information to correct creation of nitrogenase [144,145].

**Figure 1:**
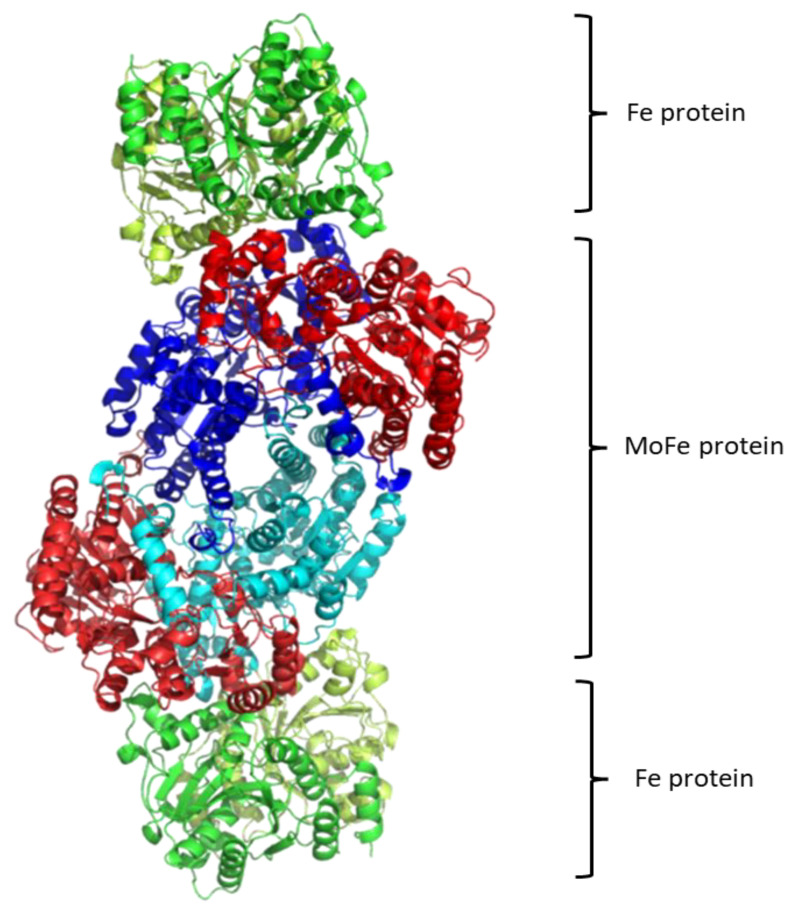
Structure of Nitrogenase complex [152].

Regarding the structure of this molecular system, see Figure 1, it contains two metalloproteins, the homodimeric iron (Fe-) protein, which is a great reductase and is responsible for the electrons’ supply. It is a dimer of two identical subunits. They are connected through two covalent bonds with one [Fe_4_S_4_] cluster [146]. (Fe-) protein is responsible for electron transfer from a reducing agent, such as ferredoxin or flavodoxin, to the nitrogenase protein (MoFe-) protein. This transfer demands an input of chemical energy. It can be covered by the binding and hydrolysis of ATP. A configuration change occurs because of the hydrolysis of ATP within the whole complex. Note that the two main metalloproteins are brought closer together so the electron transfer is easier to occur [147].

The second part of the nitrogenase complex is the heterotetrameric α_2_β_2_ or heterodimeric (αβ)_2_ molybdenum-iron (MoFe-) protein, where electrons are used for the conversion of Ν_2_ to ΝH_3_. It consists of two α and two β subunits [146]. MoFe-contains two identical iron-sulfur [8Fe-7S] clusters, namely P-clusters. They are located at the interface between the α and β subunits, counter to the other feature clusters, the two FeMo cofactors (FeMoco), which show up within the α subunits. Both subunits are of similar size and are encoded by the rifD and nifK genes [148]. The Mo cation is considered to be Mo(III), contrary to Mo(V) that prevailed earlier [149]. The [Fe_8_S_7_] core of the P-cluster consists of two cubes [Fe_4_S_3_] linked by a carbon atom. The two P-clusters are connected via covalent bonds with the rest of MoFe-through bridges that consist of six cysteine residues. Moving on to the two identical FeMo cofactors [MoFe_7_S_9_C], each contains two different clusters, i.e., [Fe_4_S_3_] and [MoFe_3_S_3_]. The last ones are linked by three sulfide ions. One cysteine and one histidine residues are used to connect each FeMo cofactor with the α submit through covalent bonds. Regarding the role of every part of the nitrogenase complex, the Fe- protein provides electrons that are entered to the P-clusters of the MoFe-protein. Then, they are transferred from the P-clusters to the FeMo cofactors, where the nitrogen fixation occurs, and the dinitrogen is connected in the central cavity of the FeMoco [144].

Some variations of this complex appear in nature. Thus, two types of such nitrogenases have been confirmed: the vanadium-iron type (VFe; *Vnf*) and the iron-iron type (FeFe; *Anf*), where the (MoFe-) protein is replaced. There are 2 α, 2 β and 2 δ or γ subunits instead of (αβ)_2_ of the usual complex [150,151]. Nevertheless, molybdenum nitrogenase, is the one that has been studied more extensively, because of its abundance versus the others and is thus the most well characterized [144]. 

#### 3.2.2. General Mechanism

As mentioned before, the reduction in N_2_ to NH_3_ demands a catalytic route to occur because of inaction of N_2_. The required activation energy for the reduction is large (*E_a_* = 230–420 kJ mol^−1^), but the enthalpy is negative (Δ*H*° = −45.2 kJ mol^−1^). This means that the whole reaction is thermodynamically favorable [153]. All these are also confirmed through the industrial fixation of N_2_ by the Haber-Bosch process, where this specific reduction takes place in temperatures ranging from 300 to 500 °C, while the pressures are more than 300 atm. The presence of Fe-based catalysts is necessary [145].

Continuing with the reduction in the substrate by nitrogenase, three basic steps occur where electrons are transfers. Firstly, the reduction in (Fe-) protein is occurred where electrons are transferred from electron carriers such as ferredoxinor or flavodoxin in vivo or dithionite in vitro to (Fe-) protein. The second step is described by the transfer of single electrons from (Fe-) to (MoFe-) protein in an MgATP-dependent process. A minimal stoichiometry of two MgATP are hydrolyzed per electron. The last e^−^ transfer occurs to the substrate which is almost certainly bound to the active site of the (MoFe-) protein [144]. The overall stoichiometry of N_2_ reduction by nitrogenase has been established as [145]:

N_2_ + 8 H^+^ + 16 MgATP + 8 e^−^ → 2 NH_3_ + H_2_ + 16 MgADP + 16 P_i_

Studying the general equation of this reaction, nitrogenase also catalyzes the reduction in H^+^ to H_2_ (which is necessary for the formation of NH_3_) along with the reduction in dinitrogen to ammonia. Additionally, it catalyzes the reduction in other small unsaturated molecules such as azide, cyanide, acetylene [154].

**Figure 2:**
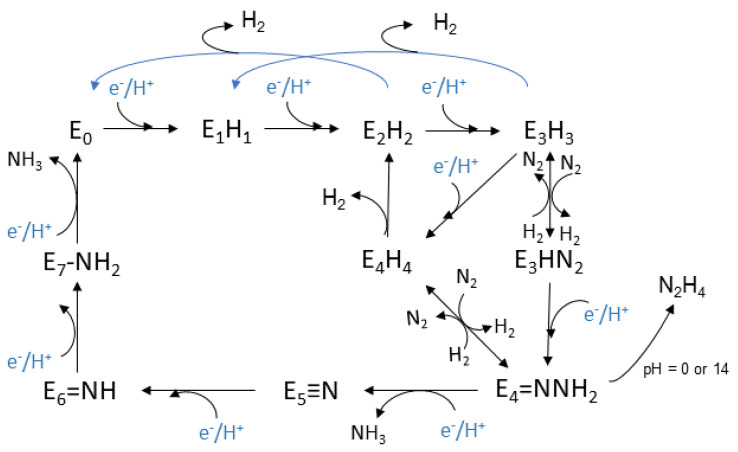
Lowe-Thorneley kinetic model [156].

The Lowe-Thorneley (LT) kinetic model, is the one that has been established for the whole process and was developed experimentally, see Figure 2. Eight H^+^ and eight e^−^ are transferred during the reaction [145,146,155,156]. Each intermediate stage is represented as *E_n_*, *n* = 0–8, which is proportionate to the numerous of the equivalents thar are transferred. The connection of N_2_ with the complex occurs at the stage *E*_4_, where four equivalents have already been transferred [146]. However, N_2_ sometimes binds to nitrogenase at the stage *E*_3_. 

This model was based on spectroscopic data that were selected throughout the process. The clarification of the mechanism is still an active area of research and a debate for the scientific community. The *E*_0_ state is the initial one where the enzyme rests at equilibrium before the catalysis begins [157]. The reductions begin at the *E*_1_ state where an e^−^ is transferred to the (Fe-) protein, with the escort of a proton (H^+^). The intermediate state *E*_2_ is described by the metal cluster being in its resting oxidation state, the two added e^−^ deposited in a bridging hydride, while the additional H^+^ is bonded to a sulfur atom. Lastly before the dinitrogen connection to the complex, the single reduced FeMo cofactor with one bridging hydride and one H^+^, belong to the *E*_3_ state. Moving on, the *E*_4_ state is considered to be a critical stage and takes part in the middle of the catalytic cycle. It appears after the accumulation of 4 pairs of electrons and protons, and it is named as Janus intermediate because of its dynamic nature. The system can decay back to E_0_, aborting the pairs that were collected or it can proceed with nitrogen binding and complete the catalytic cycle. The FeMo cofactor appears to be in its resting oxidation state with two bridging hydrides and two sulfur bonded H^+^ [145].

Based on the above intermediate states, a dynamic equilibrium is proposed for the oxidation states of the metal cluster, and especially between its initial oxidation state and a singly reduced one with additional electrons which are stored in hydrides. On the other hand, it is considered that in each step, the formation of a hydride occurs and that the metal cluster exists between the initial oxidation state and the single oxidized one [145].

**Figure 3:**
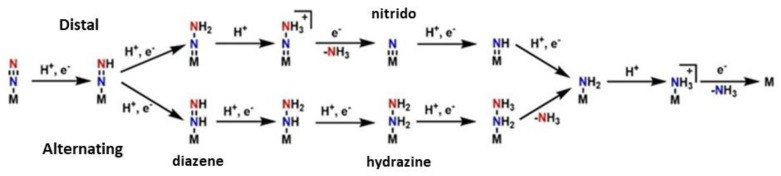
Nitrogen Fixation Mechanism [145].

Moving on towards the production of the ammonia, two basic hypotheses exist for the pathway in the second half of the mechanism: the “distal” and the “alternating” pathway, c.f. Figure 3. In the “distal” route, the dinitrogen is firstly hydrogenated on the one atom of nitrogen, leading to the release of ammonia and then the second nitrogen, which is directly bound to the metal, is hydrogenated. In the “alternating” route, the nitrogen atoms are hydrogenated alternately. This pattern goes on until NH_3_ is released from both nitrogen atoms [144,158]. It has not been clarified which pathway is correct and occurs at last. The solution to this, is the isolation of forementioned intermediates, such as the nitrido in the “distal” route and the diazene and hydrazine in the “alternating” route. However, many more problems occur from this process. The use of model complexes helps the isolation of intermediates but there is a metal center dependance. When Molybdenum model complexes are studied, the distal way predominates counter to the Iron model complexes, where the alternating pathway is preferred from the system [145].

#### 3.2.3. Calculations 

Many calculations have been performed throughout the years for this complex system and attention has been given to its catalytic role in the nitrogen fixation process. The included clusters and the cofactor have been studied and characterized independently, while there are studies of the whole complex of the metalloenzyme. Here, a review on the calculations of the states *E_n_* that were involved in the proposed mechanism is presented. 

DFT calculations have been carried out for the MoFe cofactor [MoFe_7_S_9_C], including the 35 possible broken-symmetry (BS) states in the resting state, a reduced state, and a protonated state of the cofactor. The results show that the relative energies of the calculated states depend on their geometry, the environment, i.e., surrounding protein, and the choice of the methodology, i.e., DFT functionals, basis sets. Specifically, the basis sets affect the energy values of the states, i.e., up to 11 kJ/mol. The effects of the structure of the surrounding protein result to energy differences up to 7 and 10 kJ/mol for the vdW and the electrostatic energy, respectively [159].

Single-point energy calculations using experimental geometries give similar values to the energies calculated after the optimization of geometry, but some BS states differ from the experimental ones up to 37 kJ/mol. Changing the functional from the pure TPSS to the hybrid B3LYP, a difference in energies up to 58 kJ/mol is noticed, while the correlation between the two results is small, (R^2^ = 0.57–0.72). Nevertheless, both DFT functionals are in agreement regarding the ground spin state and the reduced one. All results related to the most stable states of the structure, are useful for further calculations on the mechanism of the catalysis leading to more accurate results [159].

Furthermore, in the above study, QM/MM calculations were carried out, using the classic set Amber ff14SB force field with TIP3P for water molecules while geometry optimization was performed through TPSS-D3 method and the def2-SV(P) basis set. It was concluded that four of the Fe ions need to have the dominant α spin and three should have the opposite β spin in order to reach the experimentally observed quartet state of the cofactor, and when in asymmetric protein, there are 35 different ways that this can occur. Last but not the least, an interesting fact was concluded, namely 3 to 6 BS states of the same C3-symmetry type had close energy values leading to the fact that the protein influences a little the relative energies of the BS states that are related by the approximate three-fold symmetry of the FeMo cofactor [159].

B. Benediktsson and R. Bjornsson have carried out a series of calculations [160] where the protein environment has been taken into account. QM/MM methods are employed to study the MoFe protein and the FeMo cofactor. They concluded that only the [MoFe_7_S_9_C]^1−^ charge is a possible resting state charge. The result of −1, as a charge of the resting state, provides data in completely agreement with recent spectroscopic [161] and other computational studies [162]. Considering different spin isomers, the one that agrees with the crystallographic Fe−Fe and Mo−Fe distances has Fe cations with spin directions which lead to a rare case of spin-coupling phenomena. According to this study, on the alkoxide group on the Mo-bound homocitrate under resting state conditions, exist a proton. This proton affects the nature of the redox states of FeMoco and additionally affects some substrate reduction mechanisms [160].

Regarding the mechanism and the reaction states, the conjunction of theoretical and experimental data leads to the fact that formation of *E*_1_ is occurred via a Fe-centered reduction in combination with the protonation of a sulfide of the cluster [163]. An interesting fact about Thorhallsson and Bjornsson works [163,164] is that the used theoretical approaches for subsequent states *E_n_* (*n* = 1–8) are the same with the used ones for *E*_0_ state (CHARMM36 as a force-field for MM level of theory and TPSSh hybrid density functional for QM level, respectively). Moving on with the mechanism, it is possible for, only the *E*_0_ and *E*_1_ states to be selectively populated under conditions in which the rate of H_2_ production from the *E*_2_ state is faster than the rate of the formation of *E*_2_. Additionally, *E*_1_ models having a protonated bridging sulfide are in total agreement with the EXAFS data. All these lead to the most likely candidates to describe the *E*_1_ state. Last but not the least, minor modulation of Mo-O, Mo-Fe, and Fe-Fe distances occur throughout the process of *E*_0_ to the *E*_1_ state and the first reduction [163].

A systematic theoretical study of the relative energies of possible protonation states of the FeMo cluster in nitrogenase in the *E*_0_–*E*_4_ states has been performed via a QM/MM approach [165]. Additionally, the resting state, the states with 1–4 electrons and protons added before N_2_ binding were studied. In these calculations, the complete solvated heterotetrameric enzyme has been included for more accurate results. Two different B3LYP-D3 and TPSS-D3 dispersion corrected functionals with different basis sets, def2-SV(P) and def2-TZVPD, were used and they led to different results on the *E*_2_–*E*_4_ states, counter to the *E*_0_ and *E*_1_ states. Specifically, TPSS-D3 supports hydride ions binding to the Fe ions at the *E*_2_–*E*_4_ states creating a bridge between the Fe metals. Nonetheless, B3LYP-D3 predicts that one to three H^+^ cations are connected to the central carbide ion and that the most energetically stable structures of the *E*_2_, *E*_3_ and *E*_4_ states have the carbide ion doubly or triply protonated. Lastly, the most favorable protonation site was found to be the S2B in the *E*_1_ state [166].

The redistribution of electrons within the active site of the FeMo-co during the reductive removal of H_2_ to activate the N_2_, has also been calculated via QM/MM MD simulations. The nitrogen fixation process starts with the binding of N_2_ to *E*_4_ combined with the elimination of H_2_ [166]. This loss cannot start in absence of N_2_ in *E*_4_(4H) state, despite the fact that it interconverts with *E*_4_(H_2_,2H). This occurs because of the resulting high-energy *E*_4_(2H)* state that causes a H_2_ rebind [166]. Additionally, the non-participation of the Mo site in the electron redistribution was observed as the reaction with the N_2_ begins and it was also found that the change of Mo’s valence electrons is unlikely to occur throughout the nitrogenase cycle. Finally, it was shown that the electron redistribution upon conversion of hydride elimination and removal of H_2_ from *E*_4_(4H) to *E*_4_(2H)* is activating one or both Fe cations to bind N_2_ in the catalytically central H_2_ complex, *E*_4_(H_2_,2H). Thus, the coupled removal of H_2_ and the reduction in N_2_ is initiated [166].

**Figure 4:**
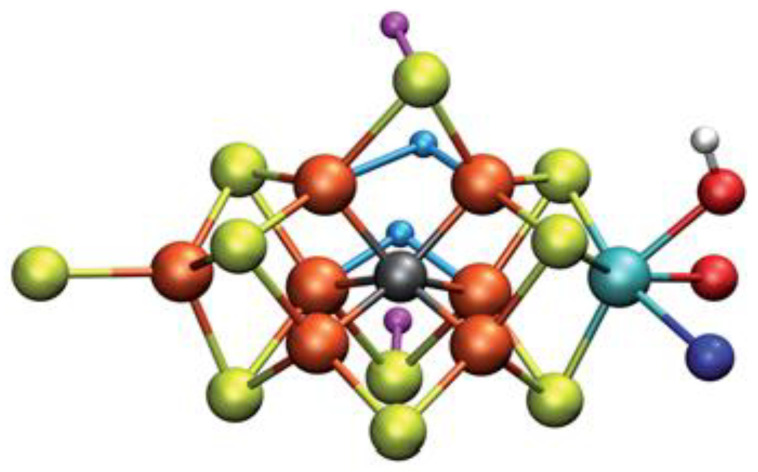
Structure of FeMo cofactor at the *E*_4_ state [164].

Specifically, the *E*_4_ state attract the research interest. Possible models for this state of nitrogenase and how N_2_ can be connected to some of these models was calculated via QM/MM approaches. Some calculations using the CHARMM36 force field for the MM approach, combined with a recent ENDOR study, result to the most favored structure of FeMo cofactor at the *E*_4_ state, see Figure 4. However, further QM calculations using hybrid functionals (B3LYP, TPSSh, M06-2X and HF exchange) lead to higher energy values for this structure counter to all open-sulfide bridge models, while this model has not been found to bind N_2_, which remains an open question to be investigated in [164]. Thorhallsson et al. proposed a mechanism for the *E*_4_ state. Specifically, the function of various components of the cofactor of nitrogenase is introduced. The cofactor’s size and the nature of the Fe–S bonds play a primary role. Moreover, the sulfide bridge between the cubanes increases the stability of the hydride. The molybdenum ion is likely to affect the redox potential of the cofactor and it could be vital for further stabilization of the N_2_-bound Fe(I) ion in *E*_4_-l-N_2_, which is formed after the reductive step, so that the N_2_ ligand to find available e^−^, to assist its activation. Finally, it has been proposed that the H^+^ on the Mo-bound alcohol group of homocitrate is in the best position, so as theN_2_ ligand to be protonated [164].

In 2020, Cao and Ryde [167] carried out a QM/MM study on N_2_ bound state of nitrogenase assuming that N_2_ is instantly protonated to a N_2_H_2_ state, and thus the issue of finding the position of the H^+^ cations in the cluster is avoided. The Amber f14SB FF was used for the protein and the MM approach and the TIP3P model was chosen to describe water molecules in the environment. The charges were obtained at TPSS/def2-SV(P) level of theory and the non-bonded model approached the metal sites. Studying both pathways, the distal and the alternating one (HNNH and NNH_2_ respectively), it was found that the binding of N_2_H_2_ is mainly occurs due to the interactions and steric clashes with the protein and not due to the intrinsic preferences of the ligand and of the cluster. Regarding the energies of the calculated states, noticeable differences are observed regarding the relative energy difference of the low-lying structures, when different functionals are used [167].

To conclude, a lot of questions are still open, like the exact way in which ligands are activated for protonation. Note that it could be very useful any additional experimental data on the *E_n_* states to further restrict the mechanistic possibilities of FeMo cofactor for comparison with the calculated data. Nonetheless, the published studies propose a pathway to clarify the mechanism of nitrogenase catalytic role [167,168,169,170,171,172,173,174,175,176,177,178].

## 4. Discussion and Conclusions

Multiscaling methodologies that combine the quantum mechanical description of specific interactions, for instance metal-ligand ones, with classical sampling of the entire system, for instance protein structure, are promising and powerful tools for computational chemistry. The studied system is split to regions. The most important area, i.e., the area where the chemical process is occurred, is calculated via a QM methodology, i.e., DFT or SE; the surrounding is studied with a less accurate method, i.e., SE or MM; while the environment with an MM approach, or via the use of a dielectric constant for the solvent, or via MD simulations. In the last case, the trajectories of the particles of the studied system are predicted. 

The commonly used QM methodology in the QM/MM and QM/MM/MD approaches is DFT which can be used in systems up to a few hundred atoms. DFT is a computational cheap methodology comparing to ab initio methods, such as multi-reference and coupled-cluster approaches, while its accuracy is comparable to them especially when the optimal functional has be used for a particular application [12,13,14,15,16,17,18,19,20,21,22,23,24,25,26,27,28,29,30,178]. B3LYP is a commonly used functional that generally works well in many applications. For more demanding applications, there is a plethora of functionals as well as many published studies that can assist for the choice of the appropriate functional. Finally, efforts are being made for the development of functionals that will be suitable for a wide range of applications [25,168]. When DFT methodology is difficult to be applied, SE methods are used. They are built on the HF formalism, but various approximations have been considered and empirical data are used. They are valuable methodologies for studying electronic effects in large molecules of biological systems and they can be applied successfully in complex systems [47,48,49,50,51,52,53,54]. When the surrounding consists of hundreds to thousands of atoms, QM (DFT or SE) calculations are not feasible, thus the potential energy of the system is defined using a force field method, where the electronic motions are ignored, and the energy of the system is calculated as a function only of the nuclear positions. Finally, MD simulations are employed to simulate system of hundreds of atoms to macromolecules of biological interest such as ribosomes, nucleosomes, metalloproteins, etc. The range of the population of atoms of the calculated systems is up to 500,000. A dynamic model is built, for instance for proteins, where the internal motions and the subsequent conformational changes significantly affect their function [59]. Algorithms are developed to calculate the trajectories through a force field approach. There are two main approaches for MD simulations: (i) the atomistic representation used for small systems and (ii) the coarse-grained method, where molecules are represented by “pseudo-atoms” approximating groups of atoms. While the first approach is more accurate, the second one is used for metalloproteins due to the size of the studied system. However, when the system is too large, i.e., liposomes with infinite radius in terms of Å, planar bilayers can be used, and thus the system can be studied via atomistic MD simulations. On the contrary, small liposomes can be fully considered using atomic level MD. Nevertheless, liposomes are generally studied better using CG models [110].

The computational study of metalloproteins and reactions involved can be a very difficult and demanding task. The presence of the metal cations that have different coordination numbers, empty or half occupied d orbitals and low lying atomic excited states further complicate the calculations. As a result, the insertion or removal of metal cations affects proteins, large conformational changes are caused, and even aggregations are formed. Thus, the study of chemical reactions of proteins and specifically: (i) the exact reaction mechanism/pathway, and (ii) the evaluation of the properties of catalytic intermediates are very hot topics.

## 5. Future Directions

The rapid increase in computer capabilities and storage in conjunction with the theory and algorithm development, increase the size of the molecular systems which can be calculated via multiscaling approaches. The most populous ones need the use of high-performance computer facilities employing QM/MM and QM/MM/MD approaches, which have been developed not only for biomolecular systems but also for modeling a variety of complex systems, i.e., inorganic/organometallic, liquids, solid-state, etc., see for instance [179,180,181,182,183,184,185,186,187,188,189,190,191].

The forthcoming step in multiscaling approaches that will lead to the increase in the size of the studied molecular systems is the use of machine learning, which is a type of artificial intelligence that trains computers to learn without being explicitly programed. It focuses on the development of suite of codes that can change when exposed to new data. Over the decades, a lot of simulations for biomolecular systems have been done with the QM/MM approach. All these data can be used to train computers to learn its own patterns. 

Finally, this review has highlighted some of the recent computational studies regarding metalloproteins, their reactions, and the interpretation of the mechanistic steps involved in nitrogenase’s complex [117,118,119,120,121,122,123,124,125,126,127,128,129,130,131,132,133,134,135,136,137,138,139,140,141,142,143,144,145,146,147,148,149,150,151,152,153,154,155,156,157,158,159,160,161,162,163,164,165,166,167,168,169,170,171,172,173,174,175,176,177]. Up to now, significant progress has been made, and details of the mechanism have been provided; however, new data on each intermediate stage of mechanism and on the excited states of the involved complexes to further restrict the mechanistic possibilities of FeMo cofactor are needed. Additionally, many questions are still unanswered, such as the exact way in which the N_2_ is activated for protonation. Future progress promises to address a lot of these questions regarding the metalloproteins’ reaction mechanisms.
